# Aberrant Retinal Pigment Epithelial Cells Derived from Induced Pluripotent Stem Cells of a Retinitis Pigmentosa Patient with the PRPF6 Mutation

**DOI:** 10.3390/ijms23169049

**Published:** 2022-08-12

**Authors:** Yuqin Liang, Feng Tan, Xihao Sun, Zekai Cui, Jianing Gu, Shengru Mao, Hon Fai Chan, Shibo Tang, Jiansu Chen

**Affiliations:** 1 Aier School of Ophthalmology, Central South University, Changsha 410015, China; 2 Aier Eye Institute, Changsha 410015, China; 3 Institute for Tissue Engineering and Regenerative Medicine, The Chinese University of Hong Kong, Hong Kong 999077, China; 4 School of Biomedical Sciences, Faculty of Medicine, The Chinese University of Hong Kong, Hong Kong 999077, China; 5 Key Laboratory for Regenerative Medicine, Ministry of Education, Jinan University, Guangzhou 510632, China

**Keywords:** retinitis pigmentosa, retinal pigment epithelium, PRPF6, disease model

## Abstract

Pre-mRNA processing factors (PRPFs) are vital components of the spliceosome and are involved in the physiological process necessary for pre-mRNA splicing to mature mRNA. As an important member, *PRPF6* mutation resulting in autosomal dominant retinitis pigmentosa (adRP) is not common. Recently, we reported the establishment of an induced pluripotent stem cells (iPSCs; CSUASOi004-A) model by reprogramming the peripheral blood mononuclear cells of a *PRPF6*-related adRP patient, which could recapitulate a consistent disease-specific genotype. In this study, a disease model of retinal pigment epithelial (RPE) cells was generated from the iPSCs of this patient to further investigate the underlying molecular and pathological mechanisms. The results showed the irregular morphology, disorganized apical microvilli and reduced expressions of RPE-specific genes in the patient’s iPSC-derived RPE cells. In addition, RPE cells carrying the *PRPF6* mutation displayed a decrease in the phagocytosis of fluorescein isothiocyanate-labeled photoreceptor outer segments and exhibited impaired cell polarity and barrier function. This study will benefit the understanding of *PRPF6*-related RPE cells and future cell therapy.

## 1. Introduction

Retinitis pigmentosa (RP) is one of the most common inherited retinal diseases characterized by progressive degeneration of retinal pigment epithelial (RPE) cells and photoreceptors [1]. The disease initially manifests as nyctalopia, followed by a gradual decrease in vision, visual field contraction and eventually blindness. The prevalence of RP is approximately one in 4000, affecting about 1.5 million individuals worldwide [2,3]. To date, mutations in more than 84 genes and loci have been identified, and 23 of them cause autosomal dominant RP (adRP; *RetNet*: sph.uth.edu/retnet, last accessed 9 June 2022). The effective treatment of RP remains a major challenge in medicine, as gene therapy for RP is limited by the high genetic heterogeneity of the disease, emphasizing the importance of studying the molecular mechanisms that are independent of mutated genes [4].

The spliceosome is an RNA and protein complex involved in the generation of mature mRNA transcripts carrying the coding protein sequence by removing non-coding introns and joining exons from pre-mRNA [5]. The spliceosome mainly consists of five small nuclear ribonucleoprotein particles (snRNPs), namely U1, U2, U4, U5 and U6. The U1 and U2 snRNPs recognize the 5′-splice site and branch site of targeted introns, respectively. The U4, U5 and U6 snRNPs are recruited to form a tri-snRNP complex [6]. Subsequently, the U1 and U4 snRNPs are released from the pre-catalytic spliceosome, and the catalytic complex is generated and activated to complete the two steps of the pre-mRNA splicing process [7]. Pre-mRNA processing factors (PRPFs) are essential for the U4/U6/U5 tri-snRNP complex, playing a critical role in the splicing process. Mutations in *PRPF* genes linked to adRP have been identified, including *PRPF3*, *PRPF4*, *PRPF6*, *PRPF8*, *PRPF31*, *SNRNP200* and *RP9* [8].

*PRPF6*, an important member of the *PRPF* family, is located on chromosome 20q13.33. More and more studies have confirmed that the PRPF6 protein not only acts as the bridge between U5 snRNP and U4/U6 snRNPs, but also promotes the formation of tri-snRNP [9,10,11]. *PRPF6* mutation can lead to adRP, while its overexpression is a common oncogenic driver of proliferation in some malignant tumors [12,13,14]. However, there is very limited published research on the pathogenesis of adRP patients with *PRPF6* mutation due to its low incidence [15]. With the advancement of molecular diagnosis, two novel missense variants, c.514C > T (p.Arg172Trp) and c.551A > G (p.Asp184Gly), were discovered in a cohort of Chinese patients with RP [16]. Still, only one variant reported in 2011, c.2185C > T (p.Arg729Trp), has been further studied [9,11,17]. Another recent manuscript showed that the expression of the mutated form of *PRPF6*, c.67C > T (p.Arg23Trp), caused mis-splicing in periventricular heterotopia disease [18]. For adRP patients with *PRPF6* mutation, it remains unclear whether some unique pathological features may predispose them to accelerated disease progression.

RPE is composed of a monolayer of polarized epithelial cells, anatomically located between the neural retina and the choroid. The RPE collaborates with the choriocapillaris and Bruch’s membrane to form the outer blood–retinal barrier, which is essential in maintaining the stability of the subretinal environment [19]. There is a rich extracellular matrix (ECM) between them. RPE cells are also responsible for the daily phagocytosis of shed outer segment membrane discs, nutrients and ions’ transepithelial transport, directional secretion of growth factors and visual cycle [20,21]. Retina degeneration is known to be a significant feature of RP, but its effect on the biological function of RPE needs to be further investigated. Induced pluripotent stem cells (iPSCs) are similar to human embryonic stem cells in their self-renewal, reproduction and differentiation potential, which can be used in disease modeling, regenerative medicine and gene therapy [22]. RPE cells can be effectively produced from iPSCs based on established protocols [23,24,25]. Regent et al. [26] developed an automated system that obtained a pure population of RPE cells without the 3-dimensional culture and manual dissection of pigmented foci during the differentiation process.

A usable disease model of adRP associated with *PRPF6* mutation plays an important role in pathogenesis research. However, such a model has not been established so far [27]. In this study, we produced a human in vitro disease model of RPE cells derived from the iPSCs of a patient carrying a pathogenic mutation in *PRPF6* (c.G2699A:p.Arg900His) (CSUASOi004-A) [28]. Then, the patient iPSC-derived RPE cells were compared with those derived from age- and sex-matched normal iPSCs. Cellular, molecular and transcriptome analyses were performed, demonstrating aberrant developmental features of the *PRPF6*-related RPE cells.

## 2. Results

### 2.1. Generation and Characterization of NC-iRPE and RP-iRPE

**Figure A1:**
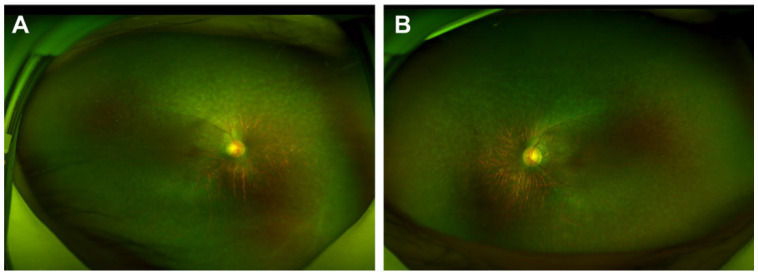
Fundus photographs of both eyes of the proband (**A**,**B**).

The proband was a 15-year-old girl who has had nyctalopia, low vision acuity and a clinical diagnosis of RP since she was a child. The fundus photographs of both eyes showed attenuated retinal arteries and extensive bone spicule pigment of the peripheral retina (Appendix A, Figure A1).

**Figure 1:**
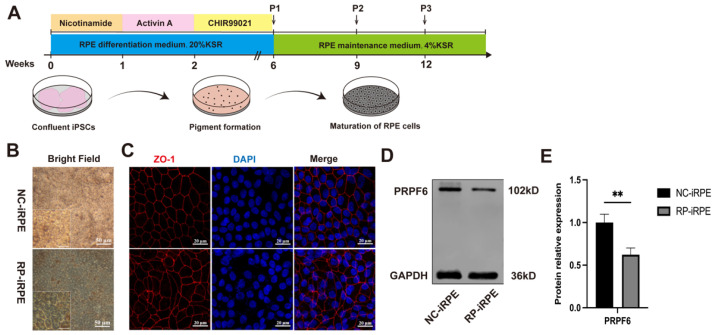
Retinal pigment epithelial (RPE) cells modeling retinitis pigmentosa (RP) from induced pluripotent stem cells (iPSCs) with *PRPF6* mutation. (**A**) Schematic diagram of differentiation procedure from normal and patient iPSCs into RPE cells (NC-iRPE; RP-iRPE). (**B**) Morphological observation of RPE cells in both groups. Scale bar 50 μm. (**C**) Immunofluorescence staining of tight junction marker ZO-1. Scale bar 20 μm. (**D**,**E**) Western blotting analysis of PRPF6 expression. GAPDH was set as the loading control. Mean ± SD (** *p* < 0.01; *n* = 3~6).

The normal and patient iPSC cell lines in this study were induced into RPE (NC-iRPE; RP-iRPE) cells according to the protocol previously established by Regent et al. [26] (Figure 1A). The morphological observation of RPE cells was determined by brightfield microscopy and immunofluorescence staining in the culture of approximately 2 weeks. Mature RPE cells derived from both iPSC cell lines displayed a classic cobblestone-like appearance, with pigmented and polygonal morphology (Figure 1B). Immunofluorescence staining of the tight junction marker ZO-1 revealed the hexagonal structure of NC-iRPE cells, while irregular, abnormal morphology was observed in RP-iRPE cells (Figure 1C). Western blotting was performed to analyze the protein expression level, showing reduced PRPF6 expression in the RP-iRPE group (Figure 1D,E).

**Figure 2:**
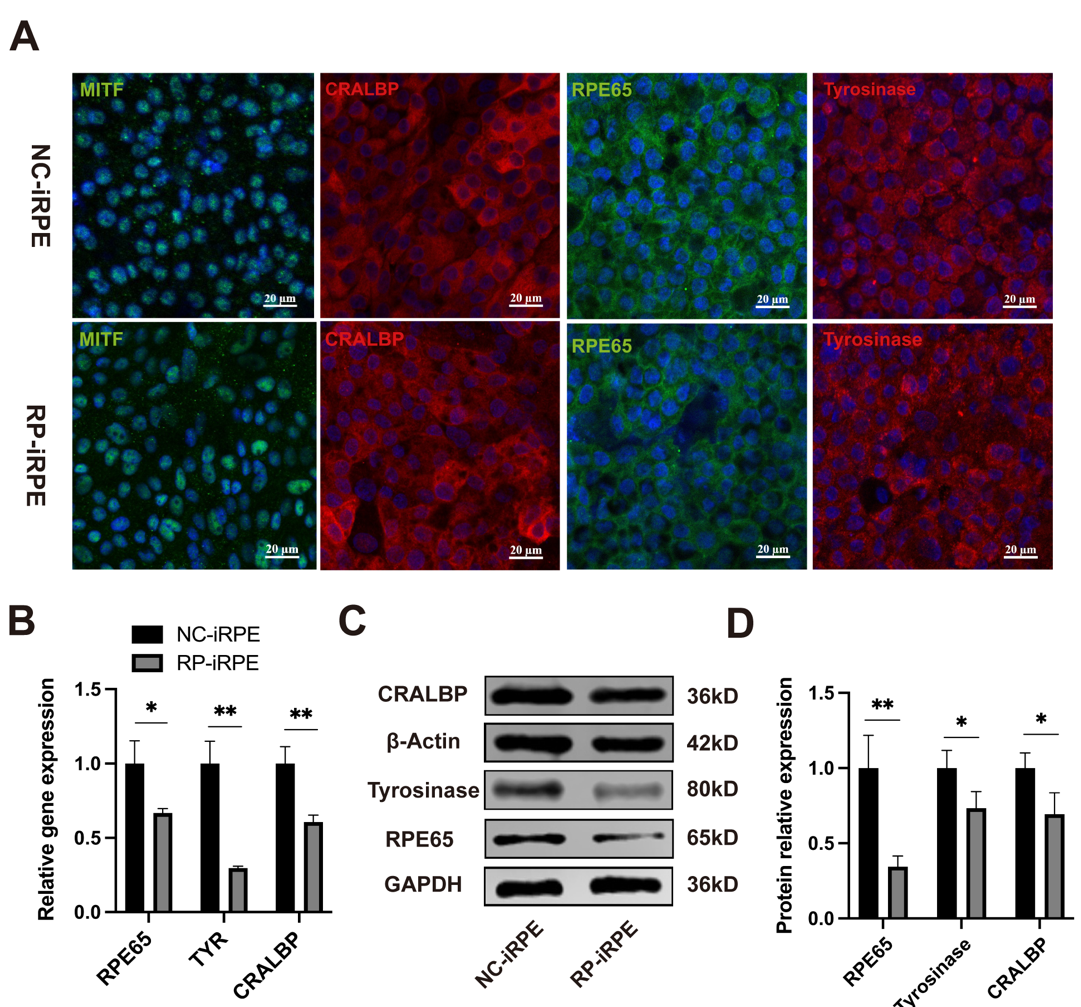
Characterization of RPE-specific gene and protein expression. (**A**) Immunofluorescence staining of MITF, CRALBP, RPE65 and tyrosinase in the NC-iRPE and RP-iRPE groups. Scale bar 20 μm. (**B**) Quantitative real-time polymerase chain reaction (PCR) results of mRNA expression for *RPE65*, *TYR* and *CRALBP*. (**C**,**D**) Western blotting analysis of RPE65, tyrosinase and CRALBP expression in both groups. GAPDH and β-Actin were set as the loading control, respectively. Mean ± SD (* *p* < 0.05; ** *p* < 0.01; *n* = 3~6).

**Figure A2:**
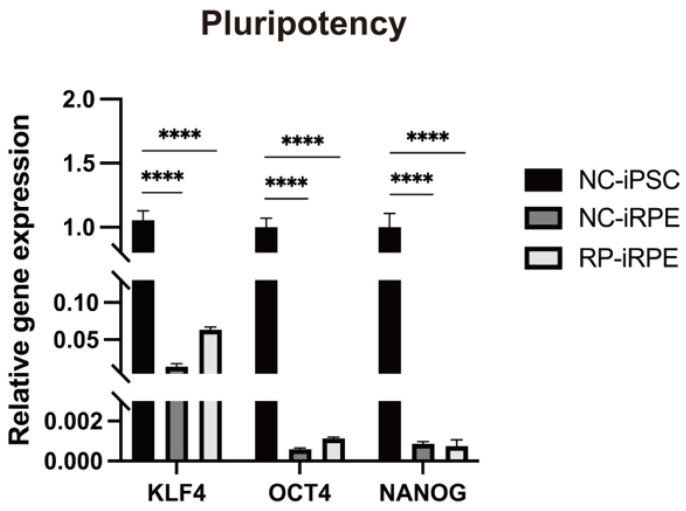
Quantitative real-time PCR results of the expression levels of pluripotency-related genes (*KLF4*, *OCT4*, *NANOG*) in the NC-iRPE group and the RP-iRPE group. Mean ± SD (**** *p* < 0.0001; *n* = 3~6).

The prerequisite for human iPSC differentiation is that the self-renewal mechanism can be switched off under the action of signaling molecules [29]. Quantitative real-time polymerase chain reaction (PCR) analysis revealed a significant decrease in the expression of pluripotency genes *KLF4*, *NANOG* and *OCT4* in mature RPE cells (Appendix A, Figure A2). Furthermore, immunofluorescence staining results showed that RPE-specific markers were expressed in both groups, including CRALBP and RPE65 as visual cycle markers, pigment synthesis marker tyrosinase and transcription factor marker MITF (Figure 2A). The mRNA expression levels of the RPE genes, *RPE65*, *TYR* and *CRALBP*, were lower in the RP-iRPE group than in the NC-iRPE group (Figure 2B). Western blotting also showed the reduced protein expressions of these genes (Figure 2C,D).

These results suggested that an adequate disease model was successfully established for subsequent research. Compared with the NC-iRPE group, the RP-iRPE group showed an irregular morphological structure and a decrease in RPE-specific markers, which indicated defective characterization in RP-iRPE cells.

### 2.2. Association of PRPF6 Mutation with Changes in RPE Cell Polarity

**Figure 3:**
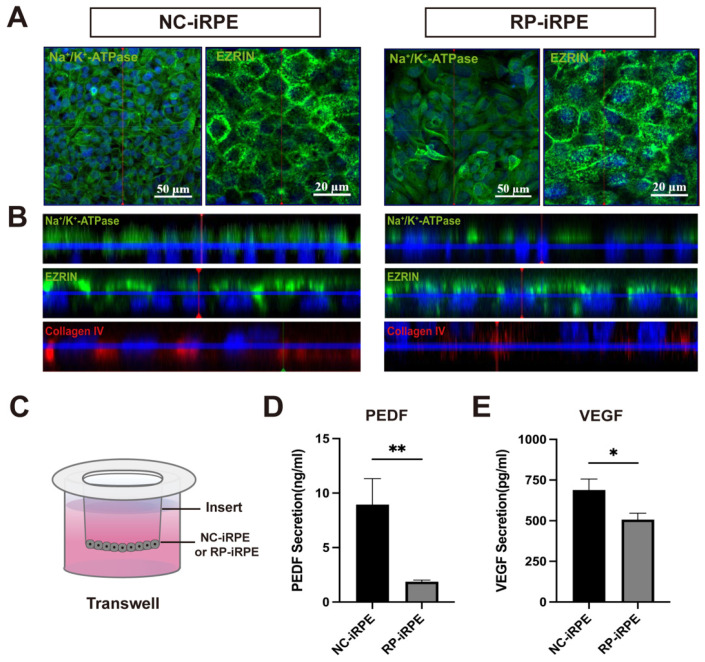
The change in polarity in RP-iRPE cells. (**A**) Immunofluorescence staining of the apical markers Na^+^/K^+^-ATPase and EZRIN. (**B**) Representative confocal z-stack micrographs showed apical Na+/K+-ATPase and EZRIN and basal collagen IV distribution in the NC-iRPE group and the RP-iRPE group. (**C**) A schematic of RPE cells seeded in a 24-transwell insert in both groups. Enzyme-linked immunosorbent assay (ELISA) assay for the apical PEDF (**D**) and basal VEGF (**E**) secretion levels, respectively. Mean ± SD (* *p* < 0.05; ** *p* < 0.01; *n* = 3~6).

**Figure A3:**
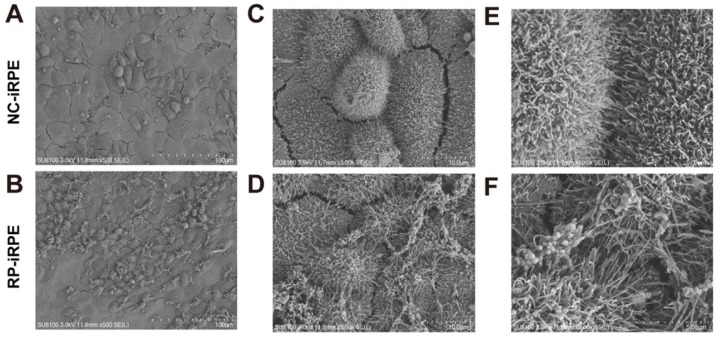
SEM showed the ultrastructural change in apical microvilli in RP-iRPE cells. Scale bar 100 μm (**A**,**B**), 10 μm (**C**,**D**), 5 μm (**E**,**F**).

The polarity of the RPE cell monolayer plays an important role in maintaining its barrier function [30]. To evaluate the expression of polarity biomarkers, immunofluorescence staining of the apical markers Na^+^/k^+^-ATPase and EZRIN was performed (Figure 3A). In addition, confocal z-stack microscopy revealed that the expression of the Na^+^/k^+^-ATPase marker in the RP-iRPE group was significantly lower than that in the NC-iRPE group, while the apically-located microvilli marker EZRIN was similarly expressed in both groups (Figure 3B). Collagen IV, the marker of basal side distribution, was reduced in RP-iRPE cells (Figure 3B). In order to understand the differences in apical microvilli between the NC-iRPE group and RP-iRPE group, an ultrastructural observation was carried out. Scanning electron microscopy (SEM) revealed that compared with the disordered microvilli structure in the RP-iRPE group, the microvilli in the NC-iRPE group were organized more neatly, and the RPE cells were more closely connected to each other without obvious intercellular space (Appendix A, Figure A3).

To further assess the highly polarized RPE monolayer, the secretions of pigment epithelium-derived factor (PEDF) and vascular endothelial growth factor (VEGF) from the apical and basal sides were measured. RPE cells derived from the NC-iRPE group and RP-iRPE group were seeded in 24-transwell inserts and cultured for 2 weeks (Figure 3C). Media samples were collected from the upper and lower chambers of the two groups. Enzyme-linked immunosorbent assay (ELISA) analysis showed that the secretion levels of PEDF and VEGF proteins in the RP-iRPE group were lower than those in the NC-iRPE group (Figure 3D,E). Together, these results revealed that the polarity was impaired in RPE cells with *PRPF6* mutation.

### 2.3. Aberrant Barrier Function in RPE Cells with PRPF6 Mutation

**Figure 4:**
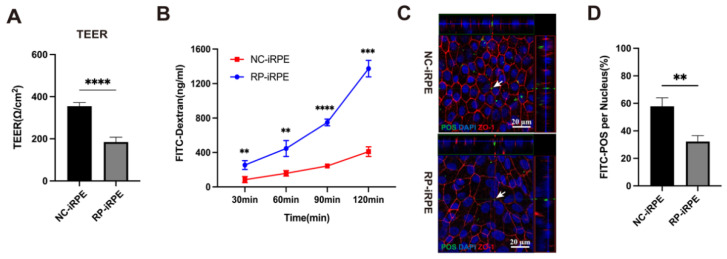
Aberrant barrier function in RP-iRPE cells. (**A**) Measurements of transepithelial electrical resistance (TEER) value showed a significant difference between the NC-iRPE group and the RP-iRPE group. (**B**) Permeability assay of the apical-to-basolateral movements at 30 min, 60 min, 90 min and 120 min after the addition of fluorescein isothiocyanate (FITC) dextran in both groups. (**C**,**D**) The phagocytosis assay shows the internalization of FITC-labeled photoreceptor outer segments (POS) (arrows) and reduced POS phagocytosis in RP-iRPE cells. Scale bar 20 μm. Mean ± SD (** *p* < 0.01; *** *p* < 0.001; **** *p* < 0.0001; *n* = 3~6).

The barrier function of RPE cells was assessed by several experiments: (1) transepithelial electrical resistance (TEER) measurement to monitor cellular dynamic barrier function; (2) permeability assay to detect the apical-to-basolateral movements of molecules; (3) photoreceptor outer segments (POS) phagocytosis test. The TEER values of the confluent RPE monolayer seeded in 24-transwell inserts achieved stability at approximately 2 weeks. The TEER values of the RP-iRPE group (184.85 ± 23.08 Ω/cm^2^, *n* = 4) were lower than those in the NC-iRPE group (355.30 ± 16.85 Ω/cm^2^, *n* = 4) (Figure 4A). Moreover, a permeability assay was performed after the stabilization of the TEER values. The concentrations of fluorescein isothiocyanate (FITC) dextran in the NC-iRPE group were 84.63 ± 35.62 ng/mL, 158.49 ± 31.99 ng/mL, 243.44 ± 11.08 ng/mL, 409.64 ± 55.40 ng/mL at 30, 60, 90 and 120 min, respectively, while they were 254.52 ± 50.77 ng/mL, 446.57 ± 92.26 ng/mL, 749.43 ± 38.91 ng/mL and 1373.60 ± 95.96 ng/mL, respectively, in the RP-iRPE group (Figure 4B). Over time, the FITC molecule penetrated from the apical-to-basal side in the RP-iRPE group more than in the NC-iRPE group. In addition to the secretion of growth factors, RPE cells are also responsible for daily POS phagocytosis, which plays an important role in maintaining the outer retinal barrier [20]. The internalized localization of POS in the two groups was observed via confocal z-stack imaging (Figure 4C, arrows). The results suggested that the number of internalized POS was markedly reduced in the RP-iRPE group (Figure 4D).

### 2.4. Transcriptome Analysis of RPE Cells with PRPF6 Mutation

**Figure 5:**
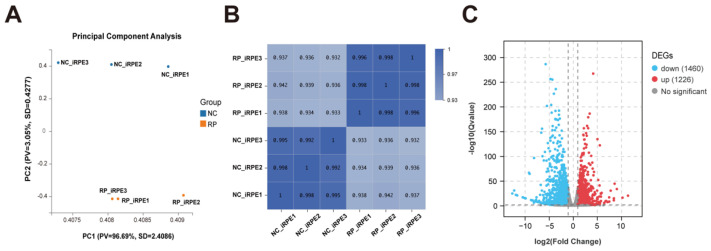
Preliminary bioinformatic analyses results of the NC-iRPE group and the RP-iRPE group. (**A**) Principal component analysis showed significant differences between the two groups of samples. (**B**) Pearson’s correlation heatmap presented high correlations within each group of samples. (**C**) The volcano plot showed the number of differentially expressed genes (DEGs) based on fold change > 2 and *p*-value < 0.05. There were 1460 downregulated genes and 1226 upregulated genes.

To investigate the potential mechanism of *PRPF6* mutation in affecting the biological functions of RPE cells, RNA-sequencing analysis was conducted between the NC-iRPE group and the RP-iRPE group. Principal component analysis (PCA) showed obvious differences between the two groups (Figure 5A). Moreover, Pearson’s correlation coefficient revealed a correlation between the biological replicates of each group (Figure 5B). A total of 2686 differentially expressed genes (DEGs) were identified based on the threshold of the adjusted *p*-value < 0.05 and fold change > 2. A volcano plot illustrated that 1460 genes were downregulated and 1226 genes were upregulated (Figure 5C).

**Figure 6:**
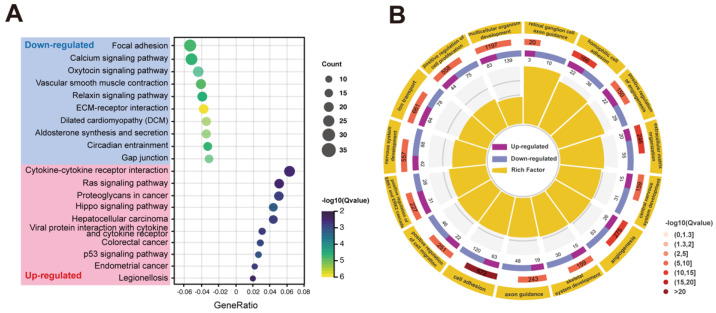
Function enrichment analysis of DEGs. (**A**) The top 20 significantly enriched signaling pathways in the RP-iRPE group from the Kyoto Encyclopedia of Genes and Genomes (KEGG) enrichment analysis were selected. (**B**) The top 15 Gene Ontology (GO) biological process terms with the largest differences between the NC-iRPE group and the RP-iRPE group.

To further explore the bioinformatic differences between the two groups, Gene Ontology (GO) terms and Kyoto Encyclopedia of Genes and Genomes (KEGG) pathways analyses were performed based on all DEGs. The top 20 KEGG signaling pathways with the most significant differences were selected (Figure 6A) (Appendix B, Table A2). Compared with the NC-iRPE group, the ECM-receptor interaction, focal adhesion, calcium signaling pathway, gap junction, circadian entrainment and aldosterone synthesis and secretion were downregulated signaling pathways in the RP-iRPE group. The upregulated signaling pathways included cytokine–cytokine receptor interaction, the Ras signaling pathway, hippo signaling pathway, hepatocellular carcinoma, p53 signaling pathway and viral protein interaction with the cytokine and cytokine receptor. Notably, ECM–receptor interaction was the most affected signaling pathway. In the GO functional enrichment, the top 15 GO terms associated with the biological process were shown with a circle diagram, including cell adhesion, extracellular matrix organization, nervous system development, the positive regulation of cell proliferation, ion transport, axon guidance and the positive regulation of the ERK1/2 cascade (Figure 6B) (Appendix B, Table A3).

**Figure 7:**
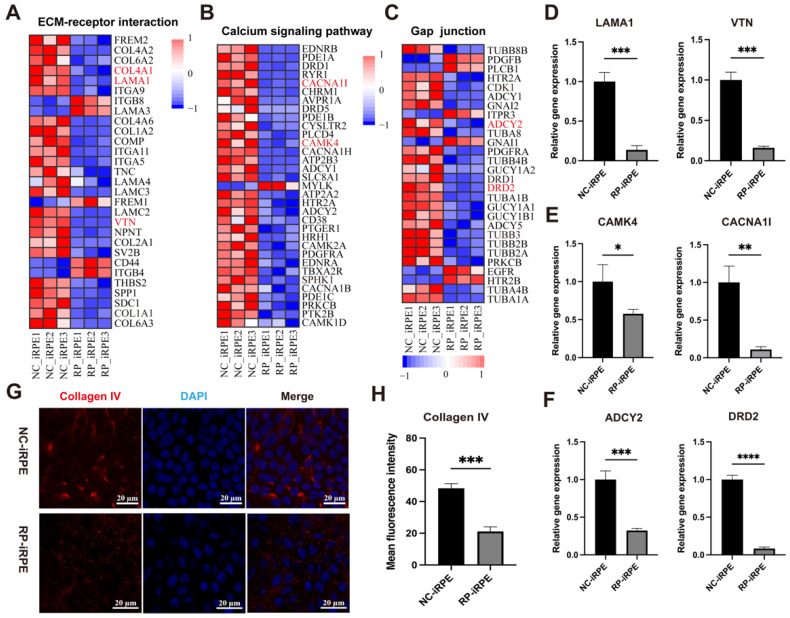
Analysis of three major signaling pathways. (**A**–**C**) The heatmaps showed the DEGs of ECM–receptor interaction, calcium signaling pathway and gap junction. (**D**–**F**) Quantitative real-time PCR results of the expressions for *LAMA1*, *VTN*, *CACNA1I*, *CAMK4*, *ADY2* and *DRD2*. (**G**,**H**) Immunofluorescence staining of collagen IV expression in the NC-iRPE and RP-iRPE groups. Scale bar 20 μm. Mean ± SD (* *p* < 0.05; ** *p* < 0.01; *** *p* < 0.001; **** *p* < 0.0001; *n* = 3).

Next, we selected three important signaling pathways that may be associated with the effects of *PRPF6* mutation on the biological function of RPE cells for further analysis based on KEGG enrichment, including ECM–receptor interaction, the calcium signaling pathway and gap junction. DEGs in these signaling pathways were obtained through RNA-sequencing data and presented in the form of heat maps (Figure 7A–C). To validate the results of transcriptome sequencing, the expression levels of the corresponding DEGs were evaluated by quantitative real-time PCR. As shown in Figure 7D–F, the expression of ECM-related genes (*LAMA1*, *VTN*), calcium signaling-related genes (*CACNA1I*, *CAMK4*) and gap junction-related genes (*ADY2*, *DRD2*) in the RP-iRPE group were significantly lower than those in the NC-iRPE group. The quantitative real-time PCR results were consistent with RNA-sequencing data. Moreover, immunofluorescence staining analysis was also performed, demonstrating lower expression of the collagen IV marker in RP-iRPE cells (Figure 7G,H).

## 3. Discussion

Despite the rapid pace of RP pathogenic gene discovery in the last decade, research into the specific pathogenesis and precise gene therapy remain as major challenges for RP due to its high genetic heterogenicity [31]. In this study, a disease model of the RPE cells derived from an adRP patient with the *PRPF6* missense variant of c.2699G > A (p.Arg900His) was successfully established, while this variant has not been previously reported in other laboratories. Moreover, we demonstrated that compared with the control, RP-iRPE cells displayed irregular morphology, disorganized apical microvilli and aberrant functional characteristics, including reduced phagocytosis of FITC-labeled POS, damaged cell polarity and barrier function. Transcriptome analysis showed that a higher ratio of downregulated DEGs in the RP-iRPE group was related to the ECM–receptor interaction, calcium signaling pathway and gap junction.

Rodent models are used to study RP associated with splicing factors by knock-in or knockout gene editing [32]. However, in addition to ethical concerns, none of the rodent models are fully representative of the onset and development of human retinal diseases due to genetic and clinical diversity [33,34,35]. For example, photoreceptors and RPE cells are the primary degenerated cells for RP, but it has been reported that splicing factors-associated animal models developed late-onset functional deficiencies in mutant RPE, while a nearly normal phenotype of photoreceptor cells was observed [36,37]. In order to overcome the translation hurdle between rodents and humans, we generated RPE cells derived from patient iPSCs that could recapitulate the genetic background of adRP caused by *PRPF6* mutation. This is beneficial for studying the correlations between the genotypes and phenotypes of the disease.

In this study, we found that RP-iRPE cells lost their classic hexagonal appearance and expressed lower levels of the RPE-specific markers RPE65, MITF, CRALBP and tyrosinase. Additionally, a decreased degree of polarity, phagocytic ability and barrier function was observed in the RP-iRPE group compared to the NC-iRPE group. Such an aberrant phenotype was also observed in iPSC-RPE cells with PRPF31 mutation [38,39]. Their results also revealed that splicing defects appeared to be correlated with the ultrastructural, cellular and functional deficiencies that are characteristic of RPE in the RP disease state [38]. Furthermore, it has previously been described that knock-in/knockout animal models of some splicing factors present an RPE degenerative phenotype [36]. A heterozygous knock-in mouse model carrying the *prpf31^p.A216P^* mutation displayed RPE pathological morphologies, including a drusen-like deposit, large lipofuscin accumulation and atrophy of basal infoldings [33]. However, intriguingly, it was reported that PRPF8 mutation caused widespread splicing changes but did not display distinctly abnormal behavior in patient iPSC-derived RPE cells [8,40]. Therefore, different member mutations from the *PRPF* family may lead to different effects on RPE. We propose the hypothesis that certain spliceosome proteins are destroyed by disease-related variants, but this has no dominant effect.

Next, RNA-sequencing was analyzed in the RP-iRPE and NC-iRPE groups. Based on the top 20 significantly enriched pathways by KEGG enrichment analysis, this study focused on ECM–receptor interaction, the calcium signaling pathway and gap junction. *COL4A1*, *LAMA1* and *VTN* were selected from the ECM–receptor interaction. Bruch’s membrane, which is rich in collagen I, collagen IV and laminin, plays a crucial role in supporting and maintaining the RPE structure and function [41]. The changes in Bruch’s membrane affect the health of the RPE and photoreceptor cells and also the onset and progression of some diseases such as RP and age-related macular degeneration [41]. Zhu et al. reported that the ECM protein vitronectin is more conducive to the in vitro culture of RPE cells compared with the laminin-521 [42]. Cruz et al. developed a therapeutic patch via the RPE monolayer immobilized on a vitronectin-coated PET membrane [43]. However, our results revealed that the expression levels of *COL4A1*, *LAMA1* and *VTN* were significantly reduced in the RP-iRPE group, which could suggest that *PRPF6* mutation affects the biological function of RPE cells by downregulating ECM-related genes. In addition, defective ECM is also associated with vascular diseases, such as cerebral cavernous malformations (CCM) [44,45,46]. Transcriptome analysis on human brain microvascular endothelial cells isolated by CCM specimens revealed that DEGs were associated with neuroinflammation, ECM remodeling, cell junction impairment and reactive oxygen species metabolism. Intriguingly, consistent with our RNA sequencing results, the ECM-related genes of *COL1A1*, *COL1A2*, *COL4A1*, *LAMA4*, *SDC1* and *TNC* were also dysregulated [46].

Calcium signaling is fundamentally important for several critical RPE functions, including the transepithelial transport of ions and water, dark adaptation of photoreceptor activity, phagocytosis and growth factor secretion [47,48]. RPE is known to embryologically originate from the neural ectoderm. *CACNA1I* and *CAMK4* have been reported to be associated with nervous development [49,50]. Moreover, the RPE cells are connected with gap junctions, tight junctions and adherent junctions [51]. *ADCY2* and *DRD2* were selected from the gap junction pathway for quantitative real-time PCR validation, and the results were consistent with transcriptomic data.

A few limitations in the present study need to be considered. The first was that only one patient was included in our research design due to the low incidence of *PRPF6*-associated adRP. Still, a greater number of patients, or/and isogenic controls generated by gene editing systems [52], will be included as far as possible in our future experiments. Meanwhile, the mechanisms by which splicing factors lead to the alterations in RPE function need more in-depth investigation due to the likely involvement of a variety of complex and comprehensive factors. Another limitation was that the interaction between photoreceptors and underlying RPE was not taken into account in our research. Mature co-culture technologies of RPE cells and retinal organoids containing photoreceptors by microfluidic chip platform will be the focus of our future efforts [53].

## 4. Materials and Methods

### 4.1. iPSCs Culture and Passaging

The patient iPSC line (CSUASOi004-A) employed in this study was generated from the peripheral blood mononuclear cells of a patient with *PRPF6* mutation, as previously reported [28]. The cells were cultured in 6-well plates coated with Matrigel (Corning, New York, USA) using mTeSR^TM^Plus medium (StemCell Technologies, Vancouver, BC, Canada) at 37 °C, 5% CO_2_ in a humidified incubator. Medium was changed every other day and enzymatically passaged with EDTA every 5 to 7 days at splitting ratios from 1:6 to 1:10. The normal iPSC cell line, as a control, was also differentiated into RPE cells under the same conditions. Sanger sequencing of all cell samples was performed regularly for genotyping verification.

### 4.2. iPSCs Differentiation to RPE Cells

RPE cells were generated from iPSCs following the previously reported method [26]. Briefly, iPSCs were grown to 80% confluence in mTeSR^TM^Plus medium and switched to RPE differentiation medium containing Dulbecco’s modified Eagle’s medium (DMEM; Gibco, Grand Island, NY, USA), 20% knockout serum replacement (KSR; Thermo Fisher Scientific, Waltham, MA, USA), 1× non-essential amino acids (NEAA; Sigma, San Luis, MO, USA), 50 μM β-mercaptoethanol (Gibco), 1% penicillin–streptomycin (Gibco) and 10 mM nicotinamide (MedChemExpress, Princeton, NJ, USA) for 0–7 days. Then, nicotinamide was replaced with 100 ng/mL Activin A (Peprotech, Rocky Hill, NJ, USA) for another week. On day 14 of differentiation, Activin A was removed from the medium, and 3 μM CHIR99021 (MedChemExpress) was added. On day 42, RPE cells were collected by incubating in TrypLE Express (Gibco) for 30 min at 37 °C and then seeded on a Matrigel-coated 6-well plate with a density of approximately 1 × 10^6^ cells/well. Meanwhile, RPE differentiation medium was replaced with RPE maintenance medium consisting of DMEM, 4% KSR, 1× NEAA, 1% penicillin–streptomycin and 50 μM β-mercaptoethanol without molecule compound. At this point, the cells were defined as passage 1 (P1). Only RPE cells at P2 and P3 were used in this study.

### 4.3. Immunofluorescence Staining

RPE cells were cultured for 14 days and washed two times with PBS. After fixing with 4% paraformaldehyde (PFA), cells were permeabilized with 0.5% Triton X-100 and blocked with 3% BSA in PBS for 1 h at room temperature. Then, cells were incubated overnight at 4 °C with primary antibodies, including mouse anti-ZO-1 (1:100, Invitrogen, Carlsbad, CA, USA), rabbit anti-MITF (1:200, Invitrogen), mouse anti-RPE65 (1:50, Abcam, Cambridge, UK), rabbit anti-CRALBP (1:100, Proteintech, Rosemont, IL, USA), rabbit anti-tyrosinase (1:100, Abcam), rabbit anti-EZRIN (1:200, GeneTex, Irvine, CA, USA), mouse anti-Na^+^/K^+^-ATPase (1:50, Santa Cruz Biotechnology, Santa Cruz, CA, USA) and mouse anti-Collagen IV (1:100, Abcam) (Appendix B, Table A1) followed by washing three times in PBS and incubation with secondary goat anti-mouse/rabbit Alexa Fluor 594/488 antibody (1:1000, Invitrogen) for 1 h at room temperature. After washing three times with PBS, cell nuclei were stained with 4′,6-diamidino-2-phenylindole (DAPI; Solarbio, Beijing, China) for 10 min. Immunofluorescence images were captured using a laser scanning confocal microscope (LSM800; Zeiss, Thornwood, Germany).

### 4.4. Reverse Transcription PCR and Quantitative Real-Time PCR

After 14 days of culture, total RNA of RPE cells was extracted with TRIzol reagent (Thermo Fisher Scientific) following the manufacturer’s instructions. HiScipt II Q RT SuperMix (Vazyme, Nanjing, China) was then used for reverse transcription of RNA into cDNA. The quantitative real-time PCR was performed using ChamQ Universal SYBR qPCR Master Mix (Vazyme) on a Roche LightCycler 96 system (Roche, Basel, Switzerland). The total volume of each reaction solution was 10 uL. The expression levels of genes were normalized to the GAPDH gene and calculated using the 2^−ΔΔCt^ method. All results were obtained from at least three independent experiments for statistical analysis.

### 4.5. Western Blotting

RPE cells were cultured for 14 days and lysed by RIPA buffer (Beyotime, Shanghai, China) for 30 min. After centrifugation, total protein concentrations of all samples were quantified using a BCA Protein Quantification Kit (Vazyme) according to the manufacturer’s protocols. Equal amounts (30 μg) of proteins per lane were loaded and separated on sodium dodecyl sulfate (SDS)-polyacrylamide gel, and then transferred to nitrocellulose membranes. After blocking with PBS containing 3% BSA for 1 h at room temperature, the membranes were incubated with primary antibodies, including rabbit anti-PRPF6 (1:1000, Invitrogen), rabbit anti-CRALBP (1:1000, Proteintech), mouse anti-RPE65 (1:1000, Abcam), rabbit anti-tyrosinase (1:1000, Abcam), mouse/rabbit anti-GAPDH (1:2000, Arigo) and mouse anti-β-Actin (1:2000, Cell Signaling Technology, Danvers, MA, USA) overnight at 4 °C. Then, membranes were incubated with goat anti-rabbit IRDye 680RD secondary antibodies or goat anti-mouse IRDye 800 CW secondary antibodies (1:10,000, LI-COR Bioscience, Lincoln, NE, USA) for 1 h at room temperature. The bands were imaged on the Odyssey Fc Imaging System (LI-COR Bioscience), and the results were analyzed by Fiji/Image J software (National Institutes of Health, Bethesda, MD, USA).

### 4.6. TEER Assay

TEER assay was used to assess the integrity of the tight junction dynamic of epithelioid cells as described previously [54,55]. RPE cells were seeded on 24-transwell inserts at a density of 1 × 10^4^ cells/insert. To test the dynamic barrier of RPE cells monolayer, TEER assay was performed every 2–3 days with a Millicell-ERS-2 volt-ohm meter (Millipore, Billerica, MA, USA) until the TEER value reached stability. The TEER value was calculated according to the following equation

TEER (Ω/cm^2^) = (R_total_ − R_insert_)/A

R_total_ is the total resistance measured (Ω), R_insert_ (Ω) is the resistance of the blank insert with media alone and A is the membrane area (cm^2^) of the insert.

### 4.7. SEM Observation

SEM was used to observe the ultrastructure of cell surface, as previously reported [56]. Briefly, RPE cells were seeded on a sterile glass slide in a 24-well plate. The slides were fixed with 2.5% glutaraldehyde (Solarbio) for 2 h at room temperature and washed gently with PBS three times. For post-fix, cells were transferred into 1% OsO_4_ (Ted Pella, Redding, CA, USA) for 1 h at room temperature and rinsed three times with PBS, and then dehydrated with a gradient concentration of ethanol (30%, 50%, 70%, 80%, 90%, 95%, 100%, 100%) and isoamyl acetate (Sigma) for 15 min each time. Finally, the samples were critical point dried, conductive metal coated and observed under a scanning electron microscope (SU8100; Hitachi, Tokyo, Japan).

### 4.8. ELISA

The media samples were collected from the upper and lower transwell chambers. The secretion levels of VEGF and PEDF protein were measured with ELISA kits (VEGF: Novus, CO, USA) (PEDF: CUSABIO, Wuhan, China) according to the manufacturer’s protocols.

### 4.9. Permeability Assay

Permeability assay was conducted by measuring the apical-to-basal movements of FITC (MedChemExpress) dextran as reported previously [57]. Briefly, cells were seeded in 24-transwell inserts. A 200 μL medium containing 100 μg/mL FITC dextran was added to the upper chamber and 1 mL medium alone in the lower chamber. In total, 200 μL of FITC dextran-treated medium was collected from the lower chamber of a 96-well plate at 30, 60, 90 and 120 min after adding the molecule; meanwhile, the same volume of fresh medium was supplemented. The fluorescence intensity of all samples at each time point was measured by a multifunctional microplate reader (Synergy HTX; BioTek, Winooski, VT, USA).

### 4.10. Phagocytosis Assay

POS were isolated from fresh porcine eyes, labeled with FITC and then applied for phagocytosis capacity of RPE as reported previously [58,59]. Briefly, POS were resuspended in 0.1 M sodium bicarbonate and incubated with FITC (MedChemExpress) in DMEM for 1 h at room temperature in the dark. After being labeled with FITC, POS were washed three times with PBS and resuspended in DMEM medium. RPE cells were incubated with FITC-labeled POS for 5 h. Then, the medium was sucked out, and the cells were washed four times with PBS. After fixing, permeabilizing and blocking, the samples were incubated with mouse anti-ZO-1 overnight at 4 °C. Cells were washed three times with PBS and incubated with goat anti-mouse Alexa Fluor 594 secondary antibody for 1 h at room temperature. After washing with PBS three times, cell nuclei were stained with DAPI for 10 min. Finally, sample images were acquired and analyzed quantitatively using a confocal microscope.

### 4.11. RNA-Sequencing Analyses

Total RNA of samples from the NC-iRPE and RP-iRPE groups were extracted using TRIzol reagent. RNA-sequencing was conducted by BGI Biotech Co., Ltd. (Wuhan, China). Raw data were filtered with SOAP [60] to remove the reads containing sequencing adaptors, low-quality and unknown bases. Next, the clean reads were mapped to the reference transcriptome sequence using Hisat2 [61] as previously described, and Bowtie2 [62] was applied to align the clean reads to the reference coding gene set. To obtain normalized gene expression levels, the reads per kilobase of exon model per million mapped reads values were calculated. The DEGs between the NC-iRPE group and RP-iRPE group were analyzed using the DESeq2 [63] with the parameters of adjusted *p* < 0.05 and fold change > 2. To gain insight into the functional enrichment of all DEGs, the GO terms and KEGG signaling pathways were performed, and the significant levels were corrected with an adjusted *p* < 0.05.

### 4.12. Statistical Analysis

Statistical analysis was performed using SPSS 26.0 software (SPSS Inc., Chicago, IL, USA) and GraphPad Prism 9.3 software (GraphPad Inc., Bethesda, MD, USA). The data were presented as mean ± SD obtained from at least three independent experiments. For a comparison between two different groups, an unpaired Student’s *t*-test was applied; comparisons among multiple groups were determined by one-way ANOVA. *p*-value < 0.05 was considered to be statistically significant.

## 5. Conclusions

In conclusion, our study confirms that the splicing factor *PRPF6* mutation affects the morphological characteristic, polarity, daily phagocytosis and barrier function of RP-iRPE cells. Long-term dysregulation of RPE functions under such conditions can cause RPE degeneration and atrophy. Our findings are helpful for understanding *PRPF6*-related RPE cells, and this study may provide a basis for gene therapy and other therapies in these patients.

## Data Availability

The data presented in this study are all contained within the main body of this article.

## Appendix B

**Table A1:** List of primary antibodies.

Antibodies	Species	Supplier	Catalog Number
ZO-1	mouse	Invitrogen	33-9100
PRPF6	rabbit	Invitrogen	PA5-61428
MITF	rabbit	Invitrogen	MA5-32554
CRALBP	rabbit	Proteintech	15356-1-AP
RPE65	mouse	Abcam	ab13826
Tyrosinase	rabbit	Abcam	ab170905
Ezrin	rabbit	GeneTex	GTX111709
Na^+^/K^+^-ATPase	mouse	Santa Cruz	sc-48345
Collagen IV	mouse	Abcam	ab86042

**Table A2:** The top 20 KEGG signaling pathways.

ID	Pathway	Gene Num	Ratio	*Q* Value
**Downregulated**				
ko04512	ECM-receptor interaction	24	0.038	4.90 × 10^−8^
ko05414	Dilated cardiomyopathy (DCM)	22	0.035	4.40 × 10^−6^
ko04925	Aldosterone synthesis and secretion	22	0.035	6.31 × 10^−6^
ko04270	Vascular smooth muscle contraction	26	0.041	1.19 × 10^−5^
ko04540	Gap junction	20	0.032	1.36 × 10^−5^
ko04510	Focal adhesion	34	0.054	1.50 × 10^−5^
ko04713	Circadian entrainment	21	0.033	1.85 × 10^−5^
ko04020	Calcium signaling pathway	33	0.052	1.96 × 10^−5^
ko04926	Relaxin signaling pathway	25	0.040	2.34 × 10^−5^
ko04921	Oxytocin signaling pathway	28	0.044	2.34 × 10^−5^
**Upregulated**				
ko04390	Hippo signaling pathway	20	0.043	5.85 × 10^−4^
ko04115	p53 signaling pathway	12	0.026	8.29 × 10^−4^
ko05210	Colorectal cancer	13	0.028	1.31 × 10^−3^
ko05213	Endometrial cancer	10	0.022	1.70 × 10^−3^
ko05205	Proteoglycans in cancer	23	0.050	1.70 × 10^−3^
ko05225	Hepatocellular carcinoma	20	0.043	1.75 × 10^−3^
ko04061	Viral protein interaction with cytokine and cytokine receptor	14	0.030	1.85 × 10^−3^
ko04060	Cytokine-cytokine receptor interaction	29	0.063	3.58 × 10^−3^
ko04014	Ras signaling pathway	24	0.052	4.27 × 10^−3^
ko05134	Legionellosis	9	0.020	4.67 × 10^−3^

**Table A3:** The top 15 GO biological process terms.

ID	Term	Gene Num	Ratio	*Q* Value
GO:0007155	cell adhesion	183	0.27	5.70 × 10^−21^
GO:0007156	homophilic cell adhesion via plasma membrane adhesion molecules	58	0.35	3.99 × 10^−12^
GO:0030198	extracellular matrix organization	75	0.30	7.64 × 10^−12^
GO:0001525	angiogenesis	79	0.29	5.73 × 10^−11^
GO:0045766	positive regulation of angiogenesis	51	0.34	2.41 × 10^−10^
GO:0007399	nervous system development	130	0.23	4.43 × 10^−10^
GO:0007411	axon guidance	67	0.28	1.02 × 10^−8^
GO:0030335	positive regulation of cell migration	68	0.27	1.71 × 10^−8^
GO:0006811	ion transport	142	0.21	2.33 × 10^−8^
GO:0031290	retinal ganglion cell axon guidance	13	0.65	1.88 × 10^−7^
GO:0007417	central nervous system development	46	0.29	4.51 × 10^−7^
GO:0008284	positive regulation of cell proliferation	119	0.21	4.83 × 10^−7^
GO:0070374	positive regulation of ERK1 and ERK2 cascade	59	0.26	6.81 × 10^−7^
GO:0007275	multicellular organism development	222	0.19	1.10 × 10^−6^
GO:0001501	skeletal system development	45	0.28	1.17 × 10^−6^
